# Antimicrobial Resistance of *Campylobacter* in Broiler Chicken Along the Food Chain in Canada

**DOI:** 10.1089/fpd.2019.2752

**Published:** 2020-08-06

**Authors:** Ousmane Dramé, Daniel Leclair, E. Jane Parmley, Anne Deckert, Blaise Ouattara, Danielle Daignault, André Ravel

**Affiliations:** ^1^ Food Safety Science Directorate, Canadian Food Inspection Agency, Ottawa, Canada.; ^2^ Groupe de recherche en épidémiologie des zoonoses et santé publique, Faculty of Veterinary Medicine, University of Montreal, Saint-Hyacinthe, Quebec, Canada.; ^3^ Centre for Foodborne, Environmental and Zoonotic Infectious Diseases, Public Health Agency of Canada, Guelph, Canada.; ^4^ National Microbiology Laboratory at Saint-Hyacinthe, Public Health Agency of Canada, Saint-Hyacinthe, Canada.

**Keywords:** antimicrobial resistance, *Campylobacter*, chicken, baseline study, Canada

## Abstract

Antimicrobial resistance (AMR) is a major public health threat worldwide. The main objective of this study was to compare AMR in *Campylobacter* from broiler chickens raised on Canadian farms and their products in different geographical regions of Canada. To do this, antimicrobial susceptibility results from isolates of *Campylobacter* recovered from a national microbiological baseline study conducted in federally registered establishments and in the retail marketplace were analyzed. Among 1460 isolates tested, 774 (53%) were resistant to at least one antimicrobial, with a predominance of three profiles: tetracycline (39%), quinolone–tetracycline (6.6%), and quinolones only (3.5%). The results showed no significant difference in the frequency of resistant profiles (*p* ≥ 0.05) among the isolates originating from different points in the food processing chain at slaughterhouses and in retail establishments. This suggests that AMR observed in *Campylobacter* isolates from raw chicken at retail originated further upstream in the system. A difference in the frequency of certain resistance profiles was observed between the regions of Canada. For instance, in British Columbia, there was more resistance to quinolones, while in Ontario and Quebec, *Campylobacter* isolates were more resistant to tetracyclines, macrolides, ketolides, and lincosamides. Comparison of AMR data from this study with those from the Canadian Integrated Program for Antimicrobial Resistance Surveillance (CIPARS) did not show any significant difference and provides evidence that CIPARS produces nationally representative resistance results.

## Introduction

Antimicrobial resistance (AMR) is a major threat to public health worldwide (Allos, 2001; Moore *et al.*, 2006; WHO, 2015). At the World Health Assembly in May 2014, the member states of the World Health Organization (WHO) supported a resolution emphasizing the urgent need for a global action plan on AMR (WHO, 2015). As a participant in the global fight against AMR, Canada also developed a new framework for action to face this challenge (PHAC, 2017).

Enteritis caused by *Campylobacter* is the second most common bacterial foodborne disease in Canada, with an estimated incidence of 447 cases per 100,000 person-years (Thomas *et al.*, 2013). In the United States, there are an estimated 310,000 drug-resistant *Campylobacter* infections per year, and 28 deaths annually are associated with this resistance (CDC, 2014). Most human cases of campylobacteriosis resolve without medical treatment, but for some patients, the use of antimicrobials to treat infections is necessary (Allos, 2001; Gibreel and Taylor, 2006; Deckert *et al.*, 2013a).

*Campylobacter* is ubiquitous in the environment and has been shown to colonize the intestinal tracts of food animals (Sahin *et al.*, 2002). Commercial poultry is an important natural reservoir of *Campylobacter jejuni*, and up to 100% of the slaughter-age broiler chickens in a single flock may harbor the organism (Sahin *et al.*, 2002). Poultry is the commodity most commonly associated with human cases of campylobacteriosis (Lindmark *et al.*, 2009; Kittl *et al.*, 2011; EFSA, 2012; Ravel *et al.*, 2017). Consumption of raw milk and untreated water is also often cited as a source of human *Campylobacter* infection (Davis *et al.*, 2016; Ravel *et al.*, 2016). Other environmental sources such as soil, manure, and aquatic environments have been identified as important contributors to human exposure to *Campylobacter* (Sahin *et al.*, 2002; Ravel *et al.*, 2017).

Antimicrobials are used for prophylaxis, treatment, or as growth promoters in food animals (Phillips *et al.*, 2004; Silva *et al.*, 2011). The Canadian Integrated Program for Antimicrobial Resistance Surveillance (CIPARS) was created in 2002 and actively monitors AMR in chickens from farms (since 2013), abattoirs, and retail sectors across Canada. CIPARS reported differences and increasing trends in AMR among retail chicken *Campylobacter* isolates across regions and over time (Agunos *et al.*, 2013).

A national microbiological baseline study (MBS) was conducted in 2012–13 across Canada with the goal of estimating the prevalence and concentration of *Campylobacter* and *Salmonella* in broiler chickens and raw poultry products processed in federally inspected abattoirs and those sold on the retail market. The present study describes the AMR profiles of *Campylobacter* isolates collected as part of the MBS. The profiles were also compared with the CIPARS data to assess the sample representativeness of CIPARS for this commodity chain.

## Materials and Methods

### Sample collection

The *Campylobacter* isolates used in this study were recovered from samples collected as part of the national MBS on broiler chickens that the Canadian Food Inspection Agency (CFIA) conducted between December 2012 and December 2013 (CFIA, 2016). The study report describes in detail the epidemiological design and sampling methodologies used to collect representative samples from the target population and products.

In brief, composite cecal samples from 20 broiler chickens from the same lot or truckload were collected during evisceration operations at the abattoir. The cecal samples were considered to be representative of farm-level *Campylobacter* contamination.

Whole chicken carcasses were collected at postchill, while carcass parts such as boneless skinless breasts and bone-in skin-on thighs were collected immediately after packing or, if not available, directly from the bulk pack container. A limited number of samples of residual liquids (exudates) were also collected from bulk packages containing 10 to 20 whole broiler chicken carcasses.

Samples of raw chicken products, including whole carcasses, boneless skinless breasts, and bone-in skin-on thighs, were also collected from supermarkets (large grocery chains) and independent grocers (including butcher shops) in Canada's 33 census metropolitan areas (CMAs).

### Isolation and speciation

Samples were tested for detection and enumeration of *Campylobacter* in accordance with the Food Safety and Inspection Service (FSIS) method MLG 41.01 (FSIS, 2011) adapted to the different matrices and described in detail in the MBS report (CFIA, 2016). Each confirmed colony type on Campy-Cefex plates was speciated using a multiplex PCR method (Health Canada, 2011, Unpublished Data) specific for *C. jejuni* and *Campylobacter coli*, and a single isolate was kept for further characterization. All available isolates of *C. jejuni*, *C. coli*, or *Campylobacter* spp. recovered from positive broiler chicken ceca and abattoir product samples were tested for antimicrobial susceptibility, as well as ∼31% of isolates recovered from retail product samples. The selection of isolates from retail products was done randomly each month.

### Antimicrobial susceptibility testing

The minimum inhibitory concentration (MIC) was determined by broth microdilution using a Sensititre Vizion™ automated system, as described by CIPARS (PHAC, 2015a). In brief, Campy plates were used to test the following nine antimicrobials belonging to seven different classes: azithromycin (AZM), ciprofloxacin (CIP), erythromycin (ERY), gentamicin (GEN), tetracycline (TET), florfenicol (FLR), nalidixic acid (NAL), telithromycin (TEL), and clindamycin (CLI). The MIC values obtained were compared with those of CLSI standards. Isolates were considered resistant if the MIC was greater than or equal to the following breakpoints: 4 μg/mL for CIP; 8 μg/mL for AZM, GEN, and CLI; 16 μg/mL for TET and TEL; 32 μg/mL for ERY; and 64 μg/mL for NAL. For FLR, only a susceptibility breakpoint of ≤4 μg/mL was available; isolates with an MIC >4 were considered resistant (PHAC, 2015a).

### Statistical analyses

Resistance to each antimicrobial tested was described by sector, region, month of sampling, and bacterial species using the R statistical software package (version 3.1.2; R Development Core Team, Vienna, Austria). In this study, we defined the resistance profile of a *Campylobacter* isolate by its response to the antimicrobials on the test panel. Thus, an isolate may have a susceptible profile (no resistance to any of the nine antimicrobials tested) or resistance profile to one or more antimicrobials.

Multivariable logistic regression was used to statistically test the association between resistance and other variables (sector of the broiler chicken supply chain, *Campylobacter* species, region, and season) for each antimicrobial separately and for resistance profiles of interest. The season variable was defined for two seasons: winter–spring (December to May) and summer–fall (June to November). For this study, the isolates for the month of November 2013 were not available at the time of AMR testing and were not included. No interactions between the variables were tested, and the probability of alpha error was set at 0.05.

For comparison with the CIPARS data, AMR data of *Campylobacter* isolates (*n* = 361) recovered from chicken ceca at slaughter and chicken legs or wings at retail from the 2013 surveillance year were used. A total of 917 isolates from the MBS were compared with those from the CIPARS; all the isolates from the processing samples collected under the MBS were excluded from the analysis. The breakdown by region was reorganized to match the CIPARS breakdown: Atlantic (New Brunswick, Nova Scotia, Newfoundland, and Prince Edward Island); Prairies (Alberta, Saskatchewan, and Manitoba); British Columbia (BC); Ontario (ON); and Quebec (QC). As no retail data from the CIPARS were available from the Atlantic region, this region was excluded for the analysis. Multivariable logistic regression models that included the source of the data were used to test for a difference between the two data sources as a function of the sector, *Campylobacter* species, region, and season for each antimicrobial separately and for selected resistance profiles.

## Results

A total of 9615 samples were collected and tested as part of the MBS, of which 7961 samples originated from slaughterhouses and 1654 from retailers (CFIA, 2016). Among the samples collected, *Campylobacter* was recovered from 24% (1025/4253) of broiler chicken lots at slaughter, 33% (1113/3343) of samples from chickens at processing, and 42% (691/1654) of samples from retail chicken. Among all the *Campylobacter* isolates recovered, 1460 were tested for antimicrobial susceptibility (Table 1). Of these1460 isolates, 1279 (87.6%) were *C. jejuni*, 176 (12.1%) *C. coli*, and 5 (0.3%) *Campylobacter* spp.

**Table 1.** Number of *Campylobacter* Isolates from Broilers and Chicken Products Tested for Antimicrobial Susceptibility by Sector Across Regions in Canada

Sector	BC	Prairie	ON	QC	Atlantic	Canada
Slaughter	220 (178;42;0)	238 (201;37;0)	174 (143;27;4)^a^	132 (123;9;0)	80 (79;1;0)	844 (724;116;4)
Processing	142 (125;17;0)	87 (76;11;0)	85 (77;7;1)	111 (106;5;0)	31 (30;1;0)	456 (414;41;1)
Retail	20 (17;3;0)	12 (11;1;0)	86 (77;9;0)	35 (30;5;0)	7 (6;1;0)	160 (141;19;0)
Total	382 (320;62;0)	337 (288;49;0)	345 (297;43;5)	278 (259;19;0)	118 (115;3;0)	1460 (1279;176;5)

^a^ Number of *Campylobacter* isolates by species (*Campylobacter jejuni*; *Campylobacter coli*; *Campylobacter* spp.).

BC, British Columbia; ON, Ontario; QC, Quebec.

### Descriptive analysis

**FIG. 1.**
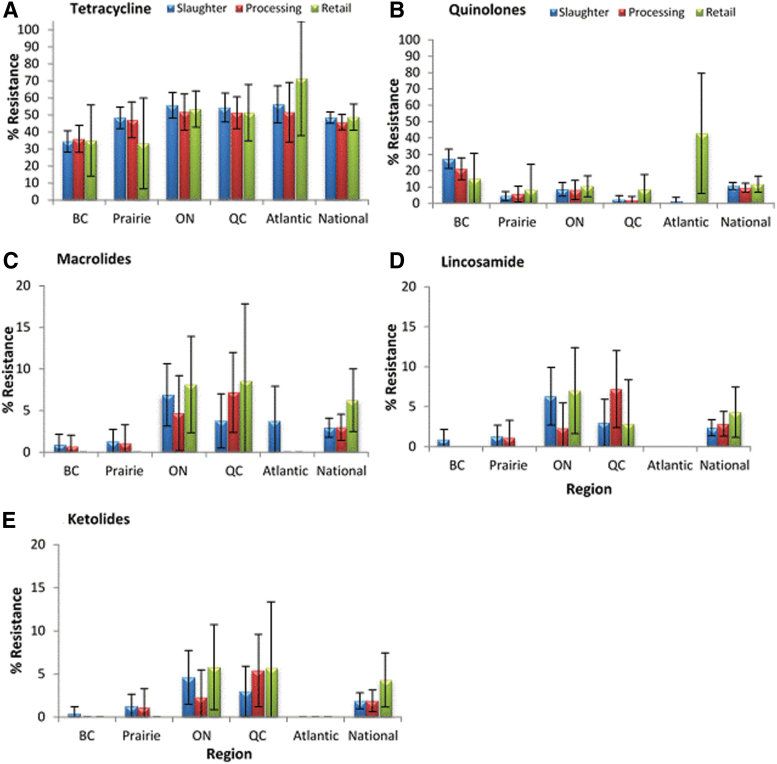
Resistance of *Campylobacter* isolated along the broiler chicken commodity chain to five antimicrobial classes. **(A)** Tetracycline; **(B)** Quinolones; **(C)** Macrolides; **(D)** Lincosamide; **(E)** Ketolides. Presented values are proportions of resistant isolates with 95% confidence interval (error bars). BC, British Columbia; ON, Ontario; QC, Quebec. Color images are available online.

Figure 1 presents the AMR by sector and by region for the different antimicrobial classes, except for phenicols (FLR) and aminoglycosides (GEN), for which there were no resistant isolates. The most common resistance was to tetracyclines (TET), and the proportion of isolates that were resistant varied between 45.8% and 48.7% nationally depending on the sector. Regionally, the lowest proportions of isolates resistant to tetracycline were observed in British Columbia (34.5% to 36%) (Fig. 1A). For the quinolones (CIP and NAL), the proportion of resistant *Campylobacter* isolates was between 10% and 12% nationally depending on the sector. Excluding the Atlantic region, where numbers (*n* = 7) are too low to be fully representative, the highest proportion of quinolone-resistant isolates was observed in British Columbia, ranging from 15% to 27%, followed by 8% to 11% in Ontario (Fig. 1B). Resistance to macrolides (AZM and ERY), lincosamides (CLI), and ketolides (TEL) displayed a similar frequency distribution pattern across the different regions (Fig. 1C–E).

The resistance profiles of *C. jejuni*, *C. coli*, and *Campylobacter* spp. are presented in Table 2. Forty-seven percent (686/1460) of all *Campylobacter* isolates were fully susceptible to all antimicrobials tested; 48.9% of *C. jejuni* isolates and 34.6% of *C. coli* isolates were susceptible. Among all the *Campylobacter* isolates that demonstrated resistance, 14 unique profiles were observed; the most common profiles were TET (39.3%), CIP-NAL-TET (6.6%), and CIP-NAL (3.5%).

**Table 2.** Resistance Profiles of *Campylobacter* Isolates Recovered from the National Microbiological Baseline Study, 2012–2013

Resistance profile	C. jejuni (*n = *1279)	C. coli (*n = *176)	Campylobacter spp. (*n = *5)	All isolates (*n = *1460)
Susceptible	625 (48.9)^a^	61 (34.6)	0	686 (46.9)
TET	517 (40.4)	56 (31.8)	1 (20)	574 (39.3)
CIP-NAL	22 (1.7)	25 (14.2)	4 (80)	52 (3.5)
CIP-NAL-TET	85 (6.6)	12 (6.8)	0	97 (6.6)
GEN-TET	1 (—^a^)	0	0	1 (—^a^)
CLI	1 (—^a^)	0	0	1 (—^a^)
AZM-ERY	3 (0.2)	0	0	3 (0.2)
AZM-ERY-TET	4 (0.3)	1 (0.5)	0	5 (0.3)
AZM-ERY-TEL-TET	1 (—^a^)	1 (0.5)	0	2 (0.1)
AZM-ERY-CLI	1 (—^a^)	7 (4)	0	8 (0.5)
AZM-ERY-CLI-TET	1 (—^a^)	0	0	1 (—^a^)
AZM-ERY-CLI-TEL	12 (0.9)	5 (2.8)	0	17 (1.1)
AZM-ERY-CLI-TEL-TET	5 (0.4)	4 (2.3)	0	9 (0.6)
AZM-ERY-CIP–NAL-CLI-TEL	0	1 (0.5)	0	1 (—)
AZM-ERY-CIP-NAL-CLI-TEL-TET	0	3 (1.7)	0	3 (0.2)

^a^ Percentage in brackets; (—) means percentage <0.01.

AZM, azithromycin; CIP, ciprofloxacin; CLI, clindamycin; ERY, erythromycin; GEN, gentamicin; FLR, florfenicol; NAL, nalidixic acid; TEL, telithromycin; TET, tetracycline.

Among the isolates that were only resistant to TET, *C. jejuni* was found to be more often resistant than *C. coli* (40% vs. 32%), whereas the opposite was observed for those that had the CIP-NAL profile (1.7% *C. jejuni* vs. 14.2% *C. coli*). Moreover, 65% of *C. coli* isolates were resistant to at least one antimicrobial, compared with 51% of *C. jejuni* isolates, which was found to be significantly different through regression analysis (Table 3). Last, three isolates, all *C. coli*, were resistant to five antimicrobial classes: macrolides, quinolones, lincosamides, ketolides, and tetracyclines (Table 2).

**Table 3.** Association Between the *Campylobacter* Resistance Profile and Species, Region, Sector, and Season According to a Multivariable Logistic Regression Analysis

Profile (number)	Compared with (number)	Species (reference* = *C. jejuni)	Region^c^ (reference* = *ON)	Sector (reference* = *Retail)	Season^*a*^ (reference = Winter)
CIP-NAL (149)	All other isolates (1306)	***C. coli*: OR = 2.8,***p* < 0.0001^b^	Atlantic: OR = 0.5, *p* = 0.2	Slaughter: OR = 0.6, *p* = 0.1	Summer = 0.9; *p* = 0.1
			Prairie: OR = 0.7, *p* = 0.2		
			**BC: OR = 4.1, *p* < 0.0001**	Processing: OR = 0.5, *p* = 0.05	
			**QC: OR = 0.4, *p* =** **0.02**^c^		
TET (691)	All other isolates (764)	*C. coli*: OR = 0.9, *p* = 0.6	**Prairie: OR = 0.7, *p* = 0.048**	Slaughter: OR = 1, *p* = 0.4	Summer: OR = 1, *p* = 0.1
			Atlantic: OR = 1, *p* = 0.9		
			**BC: OR = 0.4, *p* < 0.0001**	Processing: OR = 1, *p* = 0.6	
			QC: OR = 0.9, *p* = 0.47		
TEL (32)	All other isolates (1423)	***C. coli* OR = 7.9,***p* < 0.0001	**Prairie: OR = 0.25, *p* = 0.017**	Slaughter: OR = 0.09, *p* = 0.5	Summer: OR = 0.8, *p* = 0.5
			Atlantic: OR = 0, *p* = 0.99		
			**BC: = 0.05, *p* = 0.004**	Processing: OR = 2, *p* = 0.66	
			QC: OR = 0.8, *p* = 0.6		
AZM-ERY (49)	All other isolates (1406)	***C. coli*: OR = 9.7,***p* < 0.0001	**Prairie: OR = 0.15, *p* < 0.0001**	Slaughter: OR = 0.8, *p* = 0.5	Summer: OR = 0.8, *p* = 0.45
			Atlantic: OR = 0.6, *p* = 0.4		
			**BC:OR = 0.08, *p* < 0.0001**	Abattoir: OR = 0.9, *p* = 0.7	
			QC: OR = 1, *p* = 0.8		
CLI (40)	All other isolates (1415)	***C. coli*: OR = 11,***p* < 0.0001	**Prairie: OR = 0.15, *p* < 0.0001**^c^	Slaughter: OR = 0.9, *p* = 0.9	Summer: OR = 0.9, *p* = 0.67
			Atlantic: OR = 0, *p* = 0.99		
			**BC: OR = 0.06, *p* < 0.0001**	Processing: OR = 1.3, *p* = 0.5	
			QC: OR = 1, *p* < 0.8		
Resistance to at least one antimicrobial (769)	All fully susceptible isolates (686)	***C. coli*: OR = 2,***p* < 0.0001	**Prairie: OR = 0.6, *p* = 0.003**	Slaughter: OR = 1.1, *p* = 0.4	Summer: OR = 1.1, *p* = 0.1
			Atlantic: OR = 1, *p* = 0.8		
			**BC:OR = 0.5, *p* < 0.0001**	Processing: OR = 1, *p* = 0.6	
			QC: OR = 0.9, *p* = 0.48		
Only CIP-NAL (48)	All fully susceptible isolates (686)	***C. coli*: OR = 10.5, *p* < 0.0001**	Prairie: OR = 2.8, *p* = 0.3	Slaughter: OR = 0.8, *p* = 0.7	Summer: OR = 1, *p* = 1
			Atlantic: OR = 8, *p* = 0.09		
			**BC: OR = 20, *p* = 0.004**	Processing: OR = 0.6, *p* = 0.5	
			QC: OR = 5, *p* = 0.15		
Only CIP-NAL-TET (97)	All fully susceptible isolates (686)	*C. coli* OR = 1.4, *p* = 0.33	**Prairie: OR = 0.3, *p* = 0.004**	Slaughter: OR = 0.7, *p* = 0.25	Summer: OR = 0.9, *p* = 0.6
			Atlantic: OR = 0.3, *p* = 0.07		
			BC: OR = 1.6, *p* = 0.08	Processing: OR = 0.6, *p* = 0.15	
			**QC: OR = 0.2, *p* = 0.003**		
Only TET (573)	All fully susceptible (686)	*C. coli*: OR = 1.2, *p* = 0.3	Prairie: OR = 0.7, *p* = 0.05	Slaughter: OR = 1.3, *p* = 0.25	Summer: OR = 1.2, *p* = 0.06
			Atlantic: OR = 1.2, *p* = 0.5		
			**BC: OR = 0.3, *p* < 0.0001**	Processing: OR = 1.2, *p* = 0.35	
			QC: OR = 0.9, *p* = 0.7		

^a^ Winter corresponds to the winter–spring season (December to May) and Summer to the summer–fall season (June to November).

^b^ Bold indicates significant association (*p* < 0.05).

^c^ Regions include: BC, British Columbia; ON, Ontario; QC, Quebec; Prairie (Alberta, Saskatchewan, and Manitoba) and; Atlantic (New Brunswick, Nova Scotia, Newfoundland, and Prince Edward Island).

### Logistic regression

The statistical analysis indicated that the region and the species of *Campylobacter* had an effect (*p* < 0.05) on AMR profiles, but not the season or the sector (Table 3). Resistance observed in *Campylobacter* isolates from British Columbia and Prairies regions was often different from those in Ontario. Specifically, isolates from British Columbia and the Prairies were significantly less often resistant to all antimicrobials tested compared with Ontario, except for quinolones. There was significantly more quinolone resistance in British Columbia than Ontario and less quinolone resistance in Quebec than Ontario (Table 3). In addition, *Campylobacter* isolates from British Columbia were significantly more likely to have the CIP-NAL profile, but less likely to be resistant to one or more antimicrobials than those from Ontario.

In comparison with *C. jejuni*, *C. coli* isolates were more likely to be resistant to the quinolones and to at least one antimicrobial (Table 3). There was no significant difference in resistance to tetracycline between *C. coli* and *C. jejuni*.

### Comparison of MBS and CIPARS data

**FIG. 2.**
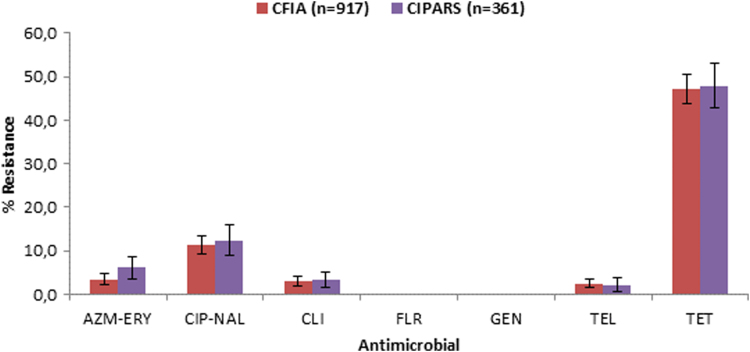
Resistance to selected antimicrobials in *Campylobacter* isolated from broiler chickens sampled along the commodity chain in 2013 according to CFIA's MBS and CIPARS data. Presented values are proportions of resistant isolates with 95% confidence interval (error bars). AZM, azithromycin; CFIA, Canadian Food Inspection Agency; CIPARS, Canadian Integrated Program for Antimicrobial Resistance Surveillance; CLI, clindamycin; ERY, erythromycin; GEN, gentamicin; FLR, florfenicol; MBS, microbiological baseline study; NAL, nalidixic acid; TEL, telithromycin; TET, tetracycline. Color images are available online.

No marked difference in AMR was observed between data from the MBS and those from the CIPARS (Fig. 2 and Table 4). The CIPARS data contained 12 resistance profiles in comparison with 10 in the MBS data, with four unique profiles for CIPARS and two unique profiles for the MBS. Three isolates, two in the MBS data and one in the CIPARS data, were resistant to five antimicrobial classes (macrolides, quinolones, lincosamides, ketolides, and tetracyclines). Approximately 11% of the isolates in the MBS data were resistant to more than one antimicrobial class, in comparison with 14% in the CIPARS data.

**Table 4.** Antimicrobial Resistance Profiles of *Campylobacter* Isolates Found in Broilers and Chicken Products According to Two Sources of Data

Resistance profile	MBS	CIPARS
Fully susceptible	431 (47.0)^a^	165 (45.7)
TET	353 (38.5)	131 (36.3)
CIP-NAL-TET	65 (7.1)	30 (8.3)
CIP-NAL	36 (3.9)	13 (3.6)
AZM-ERY-CLI-TEL	10 (1.1)	3 (0.8)
AZM-ERY-CLI-TEL-TET	8 (0.9)	3 (0.8)
AZM-ERY-CLI	6 (0.6)	3 (0.8)
AZM-ERY-TET	3 (0.3)	6 (1.7)
AZM-ERY-CIP-NAL-CLI-TEL-TET	2 (0.2)	1 (0.3)
AZM-ERY-TEL-TET	2 (0.2)	0
AZM-ERY-CIP-NAL-CLI-TEL	1 (0.1)	0
AZM-ERY	0	3 (0.8)
AZM-ERY-CIP-NAL-CLI-TET	0	1 (0.3)
AZM-ERY-CLI-TET	0	1 (0.3)
AZM-ERY-TEL	0	1 (0.3)
Total	917 (100)	361 (100)

^a^ Percentage in brackets.

AZM, azithromycin; CIPARS, Canadian Integrated Program for Antimicrobial Resistance Surveillance; CIP, ciprofloxacin; CLI, clindamycin; ERY, erythromycin; GEN, gentamicin; FLR, florfenicol; MBS, microbiological baseline study; NAL, nalidixic acid; TEL, telithromycin; TET, tetracycline.

There were no statistically significant differences between AMR data from the MBS and those from the CIPARS for the studied resistance profiles when sector, season, and region were accounted for.

## Discussion

This study showed that the AMR profiles of the *Campylobacter* isolates were similar among sectors of the broiler chicken supply chain, suggesting that resistance in *Campylobacter* found in broiler chicken meat sold at retail originated upstream, at the farm. The results of a previous study by Padungtod *et al.* (2006) on the level of resistance in isolates from chicken cecal contents and chicken meat also showed similar resistance between these two sectors. Another study, conducted in Switzerland by Kittl *et al.* (2013), demonstrated considerable overlap in the AMR level of *Campylobacter* isolates collected from chickens in abattoirs in comparison with isolates obtained from chicken meat at retail. In addition, Idris *et al.* (2006) found that fluoroquinolone resistance in *Campylobacter* can spread through an integrated commercial poultry production system from parental flocks to their progeny as both groups were colonized by the same strain of resistant *C. coli*.

Multidrug resistance is recognized as a global health problem and fluoroquinolone-resistant *Campylobacter* was identified in a WHO list of bacteria for which new antibiotics are urgently needed (WHO, 2017). The analysis of multiclass resistance showed that the proportion of isolates resistant to two or three antimicrobial classes (17%), or to four or five antimicrobial classes (1.7%), was similar to those reported by CIPARS during the same year (PHAC, 2015b). Although treatment with antimicrobials is contraindicated in most human cases of campylobacteriosis, fluoroquinolones and macrolides are commonly used when treatment is required and can be effective in reducing the duration of the illness (Deckert *et al.*, 2013b; Skarp *et al.*, 2016). The isolates resistant to the macrolide class were usually observed with resistance to other classes (results not shown). In their study done in Finland, Lehtopolku *et al.* (2010) found that *Campylobacter* isolates resistant to macrolides were uniformly multiresistant. The analysis of the AMR profiles obtained for each *Campylobacter* species indicated that *C. coli* isolates from chicken were generally more resistant to all the antimicrobial classes tested in this study (except for tetracyclines) than *C. jejuni*. This result is consistent with those of several studies (Pedersen and Wedderkopp, 2003; Padungtod *et al.*, 2006; Gallay *et al.*, 2007; Zhao *et al.*, 2010; EFSA, 2015). However, the only isolates with resistance to both quinolones and macrolides were *C. coli*, and most human infections are caused by *C. jejuni* (Friedman *et al.*, 2000).

The results of this study show a disparity in AMR among the regions of Canada. The isolates from British Columbia were more resistant to quinolones and less resistant to tetracycline compared with other regions of Canada. Tetracycline use was reported in CIPARS sentinel flocks in Ontario in 2013, but was not reported in British Columbia sentinel flocks (PHAC, 2015c). In addition, since tetracyclines have been used in animal production for many years, tetracycline resistance in populations may be impacted by historical and current use of tetracyclines, as well as coselection. A higher frequency of quinolone resistance in *Campylobacter* isolates from British Columbia was also reported by Agunos *et al.* (2013) in a study on ciprofloxacin-resistant *Campylobacter* spp. isolates from chicken at retail in Western Canada. In that study, the authors hypothesized that extralabel drug use (ELDU) of antimicrobials in veterinary medicine could be a factor contributing to the emergence of resistance in *Campylobacter*, particularly to quinolones, even though Health Canada does not recommend ELDU with drugs of Category I (Health Canada, 2008). In Canada, fluoroquinolones are approved for use in livestock to treat respiratory diseases in cattle and pigs, but none are approved for use in poultry (PHAC, 2016). According to the CIPARS report (PHAC, 2015c) on the use of antimicrobials in animals in 2013, only flocks in British Columbia reported using this class of drug. The Canadian chicken production industry took action in 2014 and eliminated the preventive use of antimicrobials considered of very high importance in human medicine (Chicken Farmers of Canada, 2019). Gallay *et al.* (2007) observed a substantial decrease in ciprofloxacin resistance in *C. jejuni* isolates from broiler chickens from 2002 to 2004, after the European Union issued recommendations limiting the use of fluoroquinolones on chicken farms. Several studies have reported that *Campylobacter* strains found in chickens from conventional farms were more resistant to ciprofloxacin than strains found in chickens from organic farms, where antibiotics were not used in production (Cui *et al.*, 2005; Luangtongkum *et al.*, 2006; Price *et al.*, 2007; Han *et al.*, 2009). In September 2005, on the basis of a risk assessment, the U.S. Food and Drug Administration (FDA) banned all use of fluoroquinolones in poultry production. Han *et al.* (2009) found that the levels of ciprofloxacin resistance in *Campylobacter* isolates from chicken at retail were 15.5% in 2004 (before the ban) and 8.5% in 2007 (after the ban). The persistence of fluoroquinolone resistance in *Campylobacter* isolates was observed in a study by Price *et al.* (2007) 1 year after a period of cessation of use in two conventional chicken operations.

The regional differences in the prevalence of AMR among the *Campylobacter* isolates in the present study could be the result of the manner in which antimicrobials are used in veterinary medicine. There are differences in production and veterinary practices between regions with regard to drug prescription, sales, or marketing since those matters are governed by provincial laws (Health Canada 2002; PHAC, 2016). Idris *et al.* (2006) found that resistance profiles for chickens reflected the drugs administered on the farm. Environmental factors that are specific to certain regions could also be a source of resistant *Campylobacter*. Messens *et al.* (2009) showed that isolates found in water lines were resistant to a number of antimicrobials administered on farms. In addition, Haruna *et al.* (2012) reported that colonization by *Campylobacter* was higher on chicken farms with a nonpotable water supply.

One of the important attributes of any health surveillance program is representativeness (Drewe *et al.*, 2015). Evaluating the representativeness of a surveillance program can be done by examining the sampling strategy or by comparing the results of the surveillance program with those obtained from a benchmarking study that is more comprehensive. The MBS was a benchmarking study and comparison of results with the CIPARS data showed that CIPARS produces nationally representative resistance results for the chicken commodity chain.
